# Electrochemical Chemically Based Sensors and Emerging Enzymatic Biosensors for Antidepressant Drug Detection: A Review

**DOI:** 10.3390/ijms24108480

**Published:** 2023-05-09

**Authors:** Renato Caldevilla, Stephanie L. Morais, Agostinho Cruz, Cristina Delerue-Matos, Fernando Moreira, João G. Pacheco, Marlene Santos, Maria Fátima Barroso

**Affiliations:** 1 CISA|ESS, Centro de Investigação em Saúde e Ambiente, Escola Superior de Saúde, Polytechnic Institute of Porto, Rua Dr. António Bernardino de Almeida, 400, 4200-072 Porto, Portugal; 10170469@ess.ipp.pt (R.C.); agostinhocruz@ess.ipp.pt (A.C.); ffm@ess.ipp.pt (F.M.); mes@ess.ipp.pt (M.S.); 2 REQUIMTE–LAQV, School of Engineering, Polytechnic Institute of Porto, R. Dr. António Bernardino de Almeida 431, 4200-072 Porto, Portugal; stephanielopesmorais@gmail.com (S.L.M.); cmm@isep.ipp.pt (C.D.-M.); jpgpa@isep.ipp.pt (J.G.P.); 3 Molecular Oncology and Viral Pathology Group, Research Center, Portuguese Oncology Institute of Porto—Francisco Gentil, R. Dr. António Bernardino de Almeida 865, 4200-072 Porto, Portugal

**Keywords:** antidepressants, chemically modified electrodes, electrochemical biosensor, enzyme-based electrochemical biosensors

## Abstract

Major depressive disorder is a widespread condition with antidepressants as the main pharmacological treatment. However, some patients experience concerning adverse reactions or have an inadequate response to treatment. Analytical chromatographic techniques, among other techniques, are valuable tools for investigating medication complications, including those associated with antidepressants. Nevertheless, there is a growing need to address the limitations associated with these techniques. In recent years, electrochemical (bio)sensors have garnered significant attention due to their lower cost, portability, and precision. Electrochemical (bio)sensors can be used for various applications related to depression, such as monitoring the levels of antidepressants in biological and in environmental samples. They can provide accurate and rapid results, which could facilitate personalized treatment and improve patient outcomes. This state-of-the-art literature review aims to explore the latest advancements in the electrochemical detection of antidepressants. The review focuses on two types of electrochemical sensors: Chemically modified sensors and enzyme-based biosensors. The referred papers are carefully categorized according to their respective sensor type. The review examines the differences between the two sensing methods, highlights their unique features and limitations, and provides an in-depth analysis of each sensor.

## 1. Introduction

### 1.1. Overview of Major Antidepressants

The World Health Organization (WHO) estimates that 280 million people suffer from depression worldwide, claiming this disease as a “major contributor to suicide deaths” [1]. The Diagnostic and Statistical Manual of Mental Disorders 5 (DSM-5) describes major depressive disorder (MDD) as a mood disorder characterized by persistent feelings of sadness, hopelessness, and loss of interest or pleasure in activities, accompanied by a range of other physical and cognitive symptoms, such as changes in appetite or sleep, fatigue, difficulty concentrating, and feelings of worthlessness or guilt, for a period of at least two weeks [2]. 

Therefore, MDD is a complex and heterogeneous illness with a complex etiopathogenesis that involves multiple factors that may act at different levels [3]. Antidepressants are the main pharmacological treatment for MDD and have been extensively used and studied for several decades now. The first antidepressants were introduced in the 1950s and since then, various classes of antidepressants have been developed with different mechanisms of action [4]. Selective serotonin reuptake inhibitors (SSRIs), serotonin-norepinephrine reuptake inhibitors (SRNIs), tricyclic antidepressants (TCAs), MAOa inhibitors (MAOaIs), and other drugs like bupropion and mirtazapine are among the main antidepressants used nowadays [5]. Although current antidepressant medications are effective and generally well tolerated, between 23% and 44% of patients under treatment experience adverse drug reactions and may drop out of the treatment [6,7,8]. Similarly, between 20% and 40% of patients respond only minimally to monotherapy [9]. Table 1 presents the most common antidepressants prescribed and their respective antidepressant class and chemical structure.

Hence, antidepressant monitorization in biological samples is extremely important for the effective and safe treatment of MDD. Moreover, antidepressants are frequently used with other drugs, and some are enzymatic inducers or inhibitors, affecting drug plasmatic levels and thus, be correlated with drug interactions or/and exacerbated adverse reactions.

In recent years, various techniques have been developed for the detection and monitoring of antidepressants in biological samples. These methods offer several advantages, such as high sensitivity and specificity, rapid analysis times, and the ability to detect multiple drugs simultaneously. By utilizing these techniques, clinicians and researchers can gain a better understanding of the pharmacokinetics and pharmacodynamics of antidepressants, improve patient outcomes, and reduce the risk of adverse drug reactions [10].

### 1.2. Traditional Analytical Techniques and Electrochemical Based (Bio)Sensing Techniques

Several analytical methodologies like gas chromatography-mass spectrometry (GC-MS), liquid chromatography-tandem mass spectrometry (LC-MS/MS), high-performance liquid chromatography (HPLC), and spectroscopy techniques (refractive index (RI), UV-Vis, fluorescence, evaporative light scattering, and NMR) are commonly used for the detection and study of different drugs. These techniques are used not only for quality control of pharmaceutical fabrication but also for drug delivery control studies, clinical toxicology, and forensic purposes [11,12,13,14]. Each of these techniques has their own advantages and has been shown to be reliable and enable the analysis and identification of practically all molecules of interest in a variety of samples, nevertheless, they all still present unresolvable problems. Normally, the sample must be remotely collected, transferred to the lab, and pre-treated prior to being analyzed. This step alone may cause the loss or decrease of several drugs due to degradation or inadequate extraction. Moreover, the sample preparation methods for these techniques can be technically challenging and time-consuming, and the data interpretation following analysis may also pose difficulties. Additionally, these methods often require expensive equipment and highly trained personnel since they may have limited portability, as well as relatively long experiment times. As a result, cheaper, rapid, and more flexible methodologies, like electrochemical based (bio)sensors, have gained popularity in the last few decades [15]. (Bio)sensors have proved to be highly effective in achieving excellent specificity for antidepressants detection, as well as allowing real-time monitoring of these drugs in the field. With their ability to detect trace amounts of antidepressants in various biological samples, these sensors have become an invaluable tool for researchers and clinicians alike. Furthermore, the real-time monitoring capability of (bio)sensors enables more timely and accurate clinical decisions and enhances patient care [11,12,13,14].

**Figure 1:**
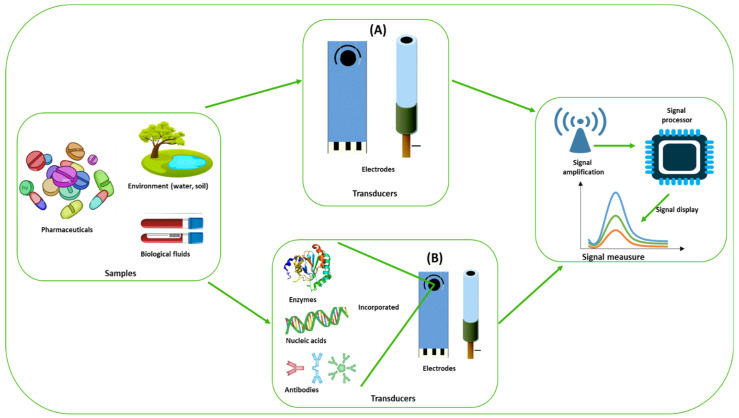
Generic scheme of an electrochemical (**A**) sensor and (**B**) biosensor.

Figure 1 depicts the main components and concepts of an electrochemical (bio)sensor. Generically, an electrochemical sensor is composed of three essential components: (i) The sample or analyte, (ii) a receptor that attaches the sample, and (iii) a transducer that converts the reaction into a measurable electrical signal. In the case of electrochemical sensors, the electrode acts as the transducer as well as the receptor. 

Electrochemical (bio)sensors require the same components as electrochemical sensors to function, but with slight modifications. In this case, (i) an analyte/target/sample with high affinity to the (ii) bioreceptor (e.g., nucleic acids, aptamers, antibodies, proteins, enzymes, peptides) attached to the (iii) transducer—electrode surface that recruits the target, which converts the binding events between target and bioreceptor into a (iv) measurable electrical signal proportional to the concentration of the target.

The analytical detection by electrochemical based (bio)sensors relies on the measurement of the electrical current generated during a chemical (e.g., redox) reaction that occurs on the surface of a working electrode. This current results from the electrolysis that occurs due to the electrochemical reduction or oxidation of the analyte. This electrochemical process depends on the mass transport rate of the analyte molecules to the electrode, as well as on the electron transfer rate at the working electrode surface. The benefits of the electrochemical approach, in contrast to the conventional analytical methodologies, include higher selectivity and sensitivity in identifying analytes, without the pre-treatment requirement. Additionally, this approach also offers other advantages, including affordability, shorter analytical times, larger linear responses, acceptable stability, higher accuracy, and good repeatability, sensitivity, as well as lower limits of detection (LOD) [15]. However, the efficient fabrication of electrochemical (bio)sensors contains some difficulties, namely in the understanding of the nuances of the adlayer formation processes and the associated fundamental electron transport mechanisms. Electrochemical measurements involve techniques based on voltammetry (cyclic, square wave, and differential pulse voltammetry), amperometry, potentiometry, and electrochemical impedance spectroscopy (EIS) [16,17].

Recently, electrochemical-based (bio)sensors have been developed for the detection and analysis of various pharmaceutical compounds, including antineoplastic drugs, anti-inflammatory compounds, antibiotics, antidepressants, etc. [18,19,20,21]. As a result of the electroactive features (e.g., capacity to be reduced or oxidized) of most antidepressants, these drugs can be detected via electrochemical procedures.

### 1.3. Aim

**Figure 2:**
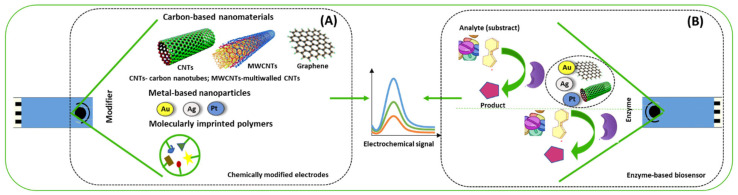
Generic scheme of an electrochemical (**A**) chemically modified sensor and (**B**) enzyme-based biosensor.

Based on the aforementioned advantages of (bio)sensors, it is crucial that more methodologies are developed for the detection of different medications, including antidepressants. Therefore, this paper highlights electroanalytical methodologies for detection/quantification of antidepressants in different matrices (environmental and biological samples). Two electrochemical approaches for this purpose are described and expanded upon this article. These methods involve the direct electrochemical assessment of antidepressants at modified electrode surfaces, with a distinction made between chemically and biologically modified electrodes (Figure 2). As indicated in Figure 2, for the chemically modified sensor (CME) construction, different chemicals are implemented in the electrode surface, while on an enzymatic biosensor, enzymes are immobilized in the electrode surface.

## 2. Chemically Modified Electrodes (CME) for the Detection of Antidepressants

In the field of electrochemistry, redox reactions take place at the electrode/solution interface, therefore, the composition of the chosen electrode must be carefully assessed, considering the repeatability of the resulting signals and the correlations of the signal-to-noise. Furthermore, the material used for the electrode design should be evaluated concerning toxicity, availability, and price. Several different materials have been used for the electrode preparation. Carbon-based materials are most often employed in electrode fabrication. Therefore, these electrodes can be fabricated with carbon from different forms and origins, namely glassy carbon, graphite, carbon fiber, boron-doped-diamond, screen printed, and carbon paste. The type of carbon utilized determines the electrochemical characteristics of the resulting electrodes [22,23].

On the other hand, metal-based electrodes are less commonly used for antidepressant monitoring, most likely due to the intense adsorption of the reactant and/or products of the redox reaction and oxide formation beneath the electrode surface, which results in the blocking of and/or change in the analytical signal, requiring surface renovation via electrochemical treatment or mechanical polishing.

Metal-based electrodes can be made from noble metals like platinum, gold, silver, rhodium, and palladium, as well as other metals, including nickel, copper, and bismuth, and can have varying sizes and geometries. The use of micrometric dimension electrodes reduces the signal-to-background ratio and lowers the detection and quantification limitations for antidepressant analysis. Very recently, many researchers have been working on the development of advanced strategies to design in vivo sensors. In this regard, biocompatible bionanomaterials have been tested to create implementable sensors in the body, namely silicon dioxide, silicon nitride, and platinum [24,25]. The understanding that a rational alteration of a conductive substrate (e.g., electrode surface) may result in electrode surfaces having the properties of both the substrate and those of the immobilized compound has cleared the path for the development of CME [26]. Considering that the modification of the electrode surfaces plays an important role in electron transfer kinetics and electrocatalytic studies, the study of CMEs has grown in popularity due to their utility in a wide range of fundamental investigations and applications.

IUPAC defines a CME as an electrode made of a conducting or semiconducting material that is coated with a selected monomolecular, multimolecular, ionic, or polymeric film of a chemical modifier and that by means of faradaic (charge-transfer) reactions or interfacial potential differences (no net charge transfer) exhibits the chemical, electrochemical, and/or optical properties of the film [27]. The substrate is the platform where the modifier is attached and selected among the electrode materials available according to the desired properties, e.g., mechanical and chemical stability, resistance to fouling, etc.

Several procedures can be performed to immobilize the modifying layer into the electrode surface, namely irreversible physical adsorption, self-assembled layers, covalent attachment, electropolymerization, entrapment in a polymer or inorganic film or incorporation into an electrode matrix [18]. The most common materials employed to prepare CME in antidepressant’s determination are carbon-based nanomaterials, metal-based nanoparticles, and molecularly imprinted polymers.

### 2.1. Carbon-Based Nanomaterials

Carbon nanotubes (CNTs) are a special group of nanomaterials since they present a unique geometry, as well as novel electrical and mechanical properties, which make CNTs a superconducting substrate, valuable for sensor development [24]. CNTs are hollow cylinders formed by rolling carbon atoms linked in hexagonal shapes with diameters lower than 100 nm. CNTs, namely single-walled carbon nanotubes (SWCNTs) and multi-walled carbon nanotubes (MWCNTs), have been used to implement electrochemical procedures for the evaluation of different antidepressants, in various samples [25,26,27,28,29,30,31].

The small nanosize and the unique optical, thermal, electrical, catalytic, magnetic, and mechanical properties of nanomaterials make them potentially useful as sensor modifiers. Nevertheless, it is difficult to control the nanomaterial based-film thickness and porosity, and its assemblage at the electrode surface needs careful supervision to achieve the desired electrode reproducibility. Amitriptyline (AMT) is a widely used tricyclic antidepressant, being the second antidepressant of its group to be approved for MDD treatment in 1961 [28,29]. AMT’s lower electroactivity at conventional electrodes paved the way for the development of a CNT-modified electrode to evaluate this antidepressant in pharmaceutical formulations. 

Using a rational mixture of CNTs and carbon paste, the electrochemical behavior of AMT presented a well-defined irreversible oxidation peak at +1.35 V vs. Ag/AgCl. Duarte et al. found that the use of sulfuric acid as an electrolyte improved AMT oxidation, possibly because of the oxidation of the alkylamine nitrogen atoms with one electron transfer and the formation of radical cations. Voltammetric techniques such as DPV, SWV, and CV were used for AMT determination achieving a LOD of 1.55 µmolL^−1^. This nano sensor was successfully applied to six different commercial pharmaceutical formulations (tablets) of this medication [29]. 

Glassy carbon electrodes (GCE) are widely used as a conductive substrate in electrochemistry since they are inexpensive, present a wide potential range, and are chemically stable. In carbon-based surfaces, such as GCE, graphite and carbon paste, it is possible to assemble different modifiers in a simple way.

Using a functionalized MWCNTs modified GCE (MWCNTs/GCE), Jat and collaborators studied the electrochemical behavior of the tricyclic antidepressant clomipramine (CMP). Similarly, to AMT, CMP also presents low redox activity. Applying this CME, it was found that CMP generated an irreversible oxidation peak at +1.120 V vs. Ag/AgCl in pH 5.5. This CME exhibited a considerable enhancement in voltammetry response, presenting a LOD of 13.2 ngmL^−1^ (41.9 nmolL^−1^). Employing voltammetric techniques, namely differential pulse anodic adsorptive stripping voltammetry (DP-AAdSV), this proposed electrochemical procedure was successfully used to quantify CMP in spiked human serum and urine samples [30]. When compared to the CNTs used as modifier, the use of MWCNTs as electrode surface modifier boosted the sensor’s sensitivity by lowering the LOD by 37-fold [29,31].

**Figure 3:**
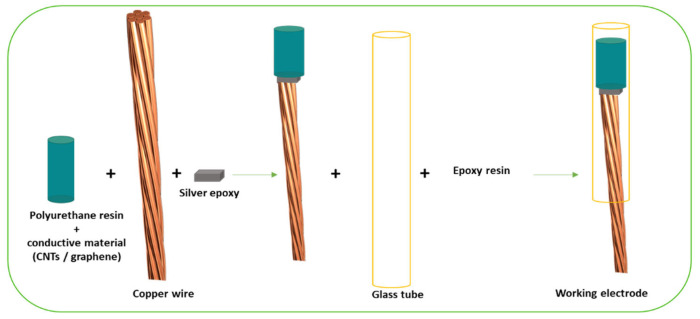
Generic scheme of composite electrode fabrication, adapted with permission from [34].

Baccarin and colleagues developed a CME (Figure 3) for escitalopram (EST) determination, a widely used SSRI, approved for MDD and other disorders like anxiety and obsessive compulsive disorder (OCD) [32,33]. Based on CV responses, the EST scanning towards positive potential revealed an irreversible oxidation process at +0.80 V (vs. SCE) when pH = 8.0. The reverse potential scans showed no indication of any reduction processes, suggesting the irreversible behavior of EST. The observed cathodic reaction of EST was attributed to the transference of two electrons in the terminal tertiary amine group. Under the optimal experimental conditions, this CME presented a linear dynamic ranges between 1.5 to 12 µmolL^−1^ and a LOD of 0.45 µmolL^−1^ with applicability to determine EST in urine and cerebrospinal fluid samples [34]. 

An electrochemical sensor for the detection of imipramine (IMP) was developed by Neto et al. The strategy followed by Neto and his team consisted of the use of a composite composed by ferrocene carboxylic acid (FCA), β-cyclodextrin (CD), and oxidized MWCNTs as modifiers on a GCE surface. The reason to use such composites was to combine the unique electronic properties of the MWCNTs, the electrochemical properties of FCA, and the ability of CD to form a complex with FCA at the electrode’s surface. Using this FCA-CD/MWCNTs-modified electrode, it was possible to analyze IMP at a very low potential (+0.02 V at pH 7.0). CV and DPV techniques were implemented, and linear ranges of 10–350 µmolL^−1^ and 0.1–10 µmolL^−1^ were obtained, respectively, enabling the detection of the antidepressant in pharmaceutical tablets and urine samples [35].

Sertraline (SRT) is one of the most widely prescribed SSRIs, marketed initially for the treatment of MDD, but is currently approved for the management of panic disorder, OCD, and post-traumatic stress disorder (PTSD) [36]. Atty et al. reported on the modification of a carbon paste rod with MWCNTs and cesium in a sodium dodecyl sulfate (SDS) medium (MWCNTs/CsCPE/SDS), capable of simultaneously detecting SRT and paracetamol in biological samples. Using CV techniques, the researchers studied the electrochemical behavior of SRT and paracetamol. An irreversible oxidation peak at +0.418 V vs. Ag/AgCl was registered for paracetamol, while an irreversible peak at +1.018 V vs. Ag/AgCl was recorded for SRT, resulting in a good separation between the two drugs and well-defined peaks, with a difference of peak potential of 0.6 V. SWV procedures were also applied to determine a LOD and limit of quantification (LOQ) for sertraline of 9.2 and 31 nmolL^−1^, respectively. The developed CME was able to detect the antidepressant and paracetamol in spiked plasma [37]. 

Trimipramine (TMP) is classified as a classical first-generation tricyclic antidepressant, with a good range of therapeutic effects in depressed patients with insomnia [38]. To enhance the sensitivity and selectivity in compound detection, different types of extraction have been applied prior to the application of detection methods. Ensafi and collaborators investigated the combination of a microporous membrane-based liquid–liquid–liquid microextraction (MM-LLLME) technique and a MWCNT-CPE to improve the selectivity and sensitivity of TMP detection. In this sense, an MM-LLLME technique, based on a polyethylene membrane saturated with isoamyl benzoate, was used to extract, purify and preconcentrate TMP from aqueous samples, thereby enhancing the sensitivity and selectivity of the CME. Utilizing this system, TMP presented an oxidation peak at +1.0 V. DPV was selected as the detection technique, estimating a limit of detection of 2$.0\;{\mathrm{nmolL}}^{-1}$. The applicability of the newly developed method was successfully verified in real plasma and urine samples, with recovery rates around 100% [39]. 

Venlafaxine (VEN) is an SNRI, approved by FDA in 1993 and widely used nowadays with good efficacy [40]. The electrocatalytic behavior of VEN was studied with CM/GCE. In this study, the chemical electrode modification was performed by using a gel containing MWCNTs and ionic liquid (IL), 1-butyl-3-methylimidazolium hexafluorophate (BMIMPF) in phosphate buffer at pH 6.8. A well-defined irreversible oxidation peak was observed at +0.780 V vs. Ag/AgCl. The electrode reaction was controlled by a diffusion process, and the redox reaction involved two electrons transferring and two protons’ participation. After the experiment conditions optimization, a linear range of 2.0 µmolL^−1^–2.0 mmolL^−1^ and a LOD of 1.69 µmolL^−1^ was determined. In the end, the proposed methodology was applied to commercial VEN capsules, with no significant differences between the proposed method and the reported conventional techniques [41]. The sensitivity of this CME was reduced when compared to the sensors produced in [31,37,39], which had a LOD 1000 times greater. Graphene is a carbon-based nanomaterial composed of a single carbon layer. In graphene, every carbon atom is linked to three adjacent carbon atoms ready to form chemical bonds. Additionally, graphene has four valence electrons. The fourth electron is free to move, enabling the graphene to conduct electricity. Graphene also has excellent semiconductor, electroactive, and transparent characteristics, with unique chemical and electrical properties that make it ideal for the development of sensor technologies [42]. In fact, graphene’s excellent ability to conduct electric charges establishes it as an excellent tool in the specific field of electrochemical biosensors [43]. Combining graphene and other electroactive materials is one of the approaches involving carbon-based materials [44,45,46].

Toledo et al. studied IMP oxidation mechanisms through electrochemical techniques, using a graphite-polyurethane composite electrode. CV allowed the establishment of the antidepressant’s voltammetric behavior, while also optimizing experimental conditions, such as pH. On the other hand, after this optimization, SWV was used to achieve a calibration curve with an IMP LOD of 4.60 nmolL^−1^. In addition, the developed method was tested in commercial pharmaceutical tablets with great success [44].

Paroxetine (PRX) is a SSRI, effective in both short-term and long-term management of depression and with efficacy in other co-morbid mental illnesses [47]. A study by Oghli and colleagues explores the fabrication of an electrochemical sensor based on a modified pencil graphite electrode (PGE). Both graphene oxide and phosphotungstic acid (GO/PWA) were used as modifiers to enhance sensitivity and catalysis of the PRX oxidation. The presence of these compounds was successfully verified through X-ray diffraction (XRD), and the modified electrode was studied by scanning electron microscopy (SEM), confirming the incorporation of GO/PWA into the PGE. Cyclic voltammograms were performed for the modified and unmodified electrodes. The CV showed an irreversible oxidation process for PRX at +1.0 V on the modified PGE.

This CME sensor exhibited a linear current response from 8.0 nmolL^−1^ to 1.0 µmolL^−1^ and a low LOD 0.9 nmolL^−1^. In addition, the influence of possible interferents was also examined, revealing the occurrence of PRX determination in the presence of probable diverse species and excipients in the pharmaceutical media. Applicability of the sensor was successfully proven by measurements in pharmaceutical tablets, urine and blood serum samples, with recovery percentages around 100% and an accurate determination of low concentrations of PRX [45].

**Figure 4:**
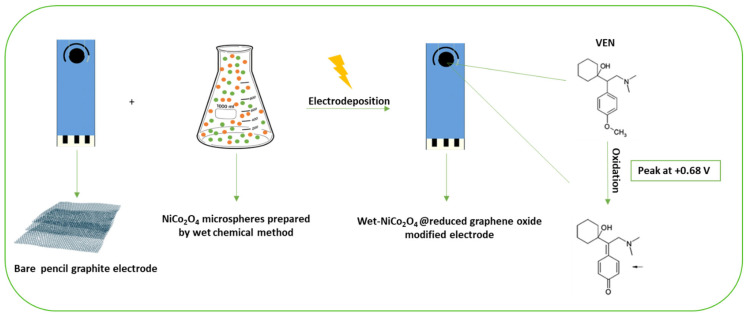
Scheme of the fabrication of a reduced graphene oxide electrochemical sensor modified by NiCo_2_O_4_ microspheres for antidepressant venlafaxine, adapted with permission from [46].

On the other hand, a study performed by Ali et al. described an additional electrochemical sensor for VEN based on the electrodeposition of nickel-cobalt oxide (NiCo_2_O_4_) microspheres (MSs), attached to a graphene oxide electrode (Figure 4). Two different deposition methods were carried out for the binary metallic oxide microstructures supported on a reduced graphene oxide (rGO) modified graphite electrode: Wet chemical and in situ-electrical deposited methods. The deposition methods of NiCo_2_O_4_ MSs were found to affect the electrochemical behavior of the modified electrodes towards the oxidation of VEN. Using the wet-NiCo_2_O_4_@rGO modified electrode, an oxidation peak was observed at +0.68 V and the peak intensity was four-times higher than in a bare PGE. Therefore, it is possible to conclude that the wet-NiCo_2_O_4_@rGO modified electrode showed the highest sensitivity in terms of the VEN oxidation peak current intensity. These results confirm the significance of the modification step’s, suitability, and sensitivity of the fabricated sensor for trace analysis of VEN in different matrices. After the characterization of the wet-NiCo_2_O_4_@rGO modified electrode and optimization of experimental parameters (pH, supporting electrolyte, etc.), SWV methods were applied to determine analytical parameters and a LOD of 3.4 nmolL^−1^ was obtained for the fabricated sensor. Furthermore, the developed method was applied to human serum samples and pharmaceutical tablets [46].

### 2.2. Metal-Based Nanoparticles

Metal and metal-organic based nanoparticles (MNPs) have many advantageous properties that make MNPs useful in the transducer component of (bio)sensors. Noble metals, such as gold, silver, platinum, rhodium, and palladium, or other metals, like nickel, copper, and bismuth NPs, have been the most popular and extensively studied. Although these noble metals are chemically inert in their macroscale form, they display unique physiochemical features at the nanoscale [48,49,50,51,52].

Due to the MNPs small size and large surface-to-volume ratio, metal NPs possess unique electrical, optical, magnetic, and catalytic properties, which makes them a promising candidate for the development of electrochemical sensors. Moreover, some metal NPs, namely gold NPs, also present biological compatibility, high binding affinity, and enhanced target selectivity [53]. Nonetheless, some of these MNPs are very expensive and toxic.

Titanium dioxide (TiO_2_) NPs are being used as electrode surface modifiers because of their high surface area, versatility, optical transparency, good biocompatibility, and relatively good conductivity. Kalanur et al. reported on a voltammetric sensor based on titanium dioxide TiO_2_-NPs, incorporated in a CPE matrix for the determination of an antidepressant used in the treatment of functional intestinal disorder: Buzepide methiodide (BZP). BZP is an organic iodide salt of buzepide and a cholinergic antagonist. A variety of electrochemical studies were carried out on BZP. Indeed, the electrochemical behavior of BZP at TiO_2_NPs-CPE indicated a quasireversible peak with an oxidation peak potential of +0.649 V and a corresponding reduction peak potential at +0.459 V. One irreversible oxidation peak of +0.945 V was also observed. TiO_2_NPs, when compared to an unmodified CPE, significantly increased the oxidation peak current of BZP, enhancing the electrochemical behavior of this analyte. After all optimized conditions were met, a linear peak current, among other characteristics, was achieved in the $50{\;\mathrm{nmolL}}^{-1}-50$ µmolL^−1^ range, detected through DPV. In addition, the most significant features of the sensor were its ability to detect BZP in the presence of a variety of interferents (ascorbic acid, glucose, sucrose, dextrose, acacia powder, starch, and talc) and its successful application on urine and human blood serum samples, with an average recovery percentages ranging from 95.8–98.8% and 89.6–97.2%, respectively [21].

A study by Tajik et al. described a nanosensor for SRT determination based on a screen-printed electrode (SPE) modified by ZnFe_2_O_4_NPs, since these have lower toxicity and higher reference daily intake values than other NPs-based materials [54]. After their synthesis, a ZnFe_2_O_4_NPs solution was added to the working electrode of the SPEs. SRT electrochemical behavior on the electrodes was then evaluated, and pH value of the sertraline concentration was optimized to 7.0. The CV scan indicated that at ZnFe_2_O_4_-SPE, SRT presented an oxidative peak at +840 mV [55], which is 178 mV less positive when compared to the CNT/CsMCPE/SDS sensor [37]. Chronoamperometric measurements of the drug at the ZnFe_2_O/SPE revealed a linear concentration range between 0.07–300 µmolL^−1^ and a LOD of 0.02 μM. Furthermore, application of the sensor to pharmaceuticals and human urine samples revealed acceptable recovery rates comparable to spectrophotometric methods [55]. When compared to the CNT-sensor [29] or MWCNTs-sensor [31,37] the ZnFe_2_O/SPE presented increased LOD (1000 fold higher).

Sultan and colleagues reported a novel electrochemical sensor, able to determine the antidepressant VEN, modified by a stable bimetallic catalyst: palladium cobalt/aluminum oxide (Co-Pd@Al_2_O_3_). The modified electrode was characterized electrochemically by CV. Square wave anodic stripping voltammetric techniques (SWAV) were applied, and an electro-oxidation response of the drug was obtained at +0.65V vs. Ag/AgCl. Using the same technique, a linear range of 1.95 nmolL^−1^–0.5 µmolL^−1^ and a LOD of 1.86 pmolL^−1^ was obtained. Additionally, the sensor was successfully applied to aqueous and serum samples, with recovery rates around 100% [56]. The utilization of a bimetallic catalyst composed of palladium cobalt/aluminum oxide increased the sensitivity of the sensor by lowering the LOD to 1.8 pmolL^−1^ when compared with the carbon-based nanomaterials where LOD are at nmolL^−1^ levels. 

In order to enhance electrochemical responses even further, recent approaches involve combining both CNTs and metallic nanoparticle modification techniques [57,58]. Using MWCNTs and mercury-modified NPs (HgNPs), Madrakian et al. developed a modified glassy carbon electrode (HgNPs/MWCNTs/GCE) capable of detecting fluvoxamine (FLV) in body fluids. 

The doubly modified electrodes revealed, by CV techniques, intensity values of cathodic peak currents for FLV 2 to 3-fold higher than the GCE modified by either only MWCNTs or mercury nanoparticles. In fact, with a bare GCE, FLV did not present any electrochemical behavior. However, at HgNPs/GCE, MWCNTs/GCE, and HgNPs/MWCNTs/GCE, FLV presented electrocatalytic activity, via a reduction process. At the HgNPs/GCE, MWCNTs/GCE, and HgNPs/MWCNT/GCE reduction peaks appeared at approximately −0.87, −0.82, and −0.76 V vs. Ag/AgCl, respectively, and at the oxidation step, no oxidation peak was observed. After optimization of the experimental conditions, DPV techniques were able to determine a LOD of 0.01 µmolL^−1^. The novel sensor was applied to human urine samples and pharmaceutical tablets, with recovery rates of 96%, high sensitivity, and reproducibility [57].

Shoja et al. reported the development of a modified GCE with gold NPs (AuNPs), enriched with MWCNTs and an electropolymerized nanostructured levodopa film (NiLD/AuNPs/MWCNTs/GCE) for the detection of SRT. After studying the electrochemical behavior of the CME electrode and SRT through CV and DPV techniques determining a linear range of 0.05–5.50 mmolL^−1^ for the compound was used. Furthermore, the sensor was successfully applied to human serum samples [58].

### 2.3. Molecularly Imprinted Polymers

In the last years, the integration of molecularly imprinted polymers (MIPs) into different sensing devices to detect measurable signal after achieving selective molecular binding has been widely studied as an alternative to natural biological elements. MIPs are one of the most promising tools for the design and construction of synthetic biomimetic recognition systems, since they are analogs of natural antibody-antigen systems [59].

This has allowed the development of several MIP based sensors for detection of a wide range of analytes with applications in different matrices. Among several transducers (optical, mass), the combination of MIPs with electrochemical transduction has attracted attention due to their simplicity, ease of preparation, versatility, and ease of miniaturization. 

MIPs are typically easy to prepare, reusable, cheap, and can be prepared to selectively recognize the target molecule of interest. They are resistant to different changes in chemical and thermal conditions, which is an advantage when compared to natural receptors. 

**Figure 5:**
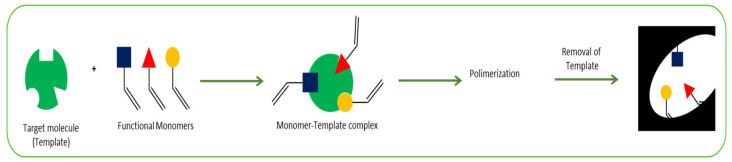
Summary of the MIPs preparation procedure.

The most common procedure for MIP synthesis is chemical polymerization. Initially, the target molecule is mixed with functional monomers in the presence of a cross-linking agent and a porogenic solvent. Then, a polymerization reaction occurs, triggered by an initiator (thermal or photo initiated), and a polymer with the target molecule incorporated within it is obtained. The last step is the removal of the imprinted molecules from the polymer matrix, normally using solvent extraction, producing specific binding sites which are complementary in size, shape, and functionality to the target analyte (Figure 5). This allows the obtention of a highly selective polymer. A control designated non-imprinted polymer (NIP), under the same conditions but without the presence of the analyte, is normally prepared to validate the selectivity [60,61].

The key factor in MIP preparation is the monomer choice since its selectivity is strongly dependent on the integration between the monomer and the template. The stronger the integration in the pre-polymerization solution, the better the recognition sites are obtained. Typically, monomer selection is achieved by trial and error. However, recently computer modeling studies have proven to be an excellent solution to aid in the selection of the monomer, reducing the amount of experiments and reagents [62,63,64]. Two types of interaction between template and functional monomer can be used, namely covalent and non-covalent approaches. Covalent imprinting is based on the use of reversible reactions, allowing a strong interaction and the obtention of highly specific binding sites. Still, the number of reactions available is limited and the approach leads to slow binding and the removal of templates. Therefore, non-covalent imprinting, where integrations are based on ionic interactions, van der Waals forces, π-π interactions, and hydrogen bonding, is the most used approach. With this, the removal and rebinding of the analyte are simpler and faster [60].

MIP preparation can be obtained with different procedures and imprinting techniques. Free-radical polymerization is the most common reaction utilized in conjugation with different techniques such as bulk, emulsion, suspension, seed, and precipitation polymerization. The choice of the imprinting technique is related with the type of particles to be obtained and the type of application to be used. In the preparation of electrochemical sensors, a special type of preparation, electropolmerizing has been widely used and proved to be an excellent choice for this kind of sensor. It is based on the use of a monomer that can be polymerized by the passage of current. It is a very simple and fast method. The polymerization occurs in situ on the working electrode’s surface. It can reduce the number of reagents used, and polymers with different conductivities can be obtained. The film thickness can be easily controlled by changing the current, which traduces into good reproducibility. Another strategy to modify nanoparticles is to synthesize MIPs and combine them with NPs or CNTs.

Fluoxetine (FLU) is an SSRI that increases the concentration of 5-hydroxytryptamine(5-HT) in the brain areas without affecting other neurotransmitter receptors [65]. Alizadeh and colleagues described a highly promising graphene electrochemical sensing platform, molecularly imprinted polymer-modified CPE (graphene MIP-modified CPE) for the determination of FLU. The synthesis of the MIP was conducted through the precipitation polymerization method. Moreover, methacrylic acid and vinyl benzene (VB) were used as functional monomers. Graphene augmenting characteristics were evaluated through the incorporation of the material in the nano-MIP-modified CPE by two different methods. While in the first method, graphene was mixed with graphite and nano-MIP (nano-MIP/G1-CP), the second involved the addition of the compound and nano-MIP to dimethyl formamide (nano-MIP/G2-CP). Nano-MIP/G2-CP showed the most potential in terms of optimal DPV results. Selectivity of the proposed sensor was evaluated through the comparison with other drugs (FLU, trifluoperazine, clozapine, oxazepam, and salbutamol), with DPV values significantly higher for FLU. The sensor was successfully used for the detection of FLU in both plasma and pharmaceutical detection assays, with recovery percentages ranging from 91.5 to 110% [66].

**Figure 6:**
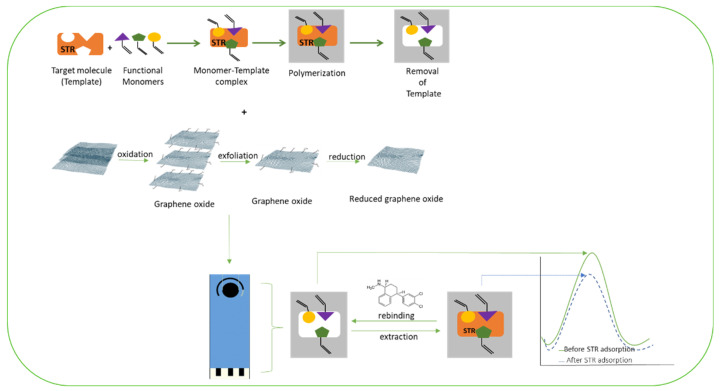
Schematic representation of the modification of a screen-printed carbon electrode by MIP and graphene oxide nanosheets for determination of sertraline, adapted with permission from [67].

A paper by Khosrokhavar et al. reported a screen-printed carbon electrode (SPCE) altered by MIP nanoparticles and embedded with graphene suspension. The researchers evaluated the electrochemical performance of 3 different types of electrodes: the bare SPCE, SPCEs embedded with graphene (Gr-SPCE), and SPCEs modified by both the MIP and graphene (MIP/Gr-SPCE). K_3_[Fe(CN)_6_] reduction to K_4_[Fe (CN)_6_] was considered as the redox couple used in the DPV and CV methods. MIP/Gr-SPCEs exhibited greater intensity in voltammetric signals when compared to the other electrodes. The MIP/Gr-SPCEs also showed a much higher response to SRT, which suggests that the specific imprinting sites for SRT in the MIP are a major factor in potentiating the sensor’s capabilities (Figure 6). A linear response between 5.0 nmolL^−1^–0.75 µmolL^−1^ was determined, and the novel sensor was successfully applied to pharmaceuticals and human serum samples, revealing its ability to detect the drug in a variety of complex samples [67].

A CPE, combined with MWCNT’s and MIP selective for TMP, was fabricated and reported by Akhoundian et al. The MIPs were synthesized via precipitation polymerization method. A mixture of MWCNT and graphite was added to the TMP-MIP, and the resulting compound was added to the electrodes. Conditions such as the pH of the TMP solution, extraction solution volume and extraction time were all optimized. The sensor revealed a linear response range of 0.1–25 nmol$L^{-1}$, very high sensitivity (2131 µA µ${\mathrm{molL}}^{-1}$), and good selectivity, when compared with other molecules (nortriptyline, IMP, and AMT), confirmed through application on blood and serum samples [68].

Trazodone (TZD) is an antidepressant drug that belongs to the class of serotonin antagonists and reuptake inhibitors. Isabel et al. described a disposable voltammetric MIP based sensor for TZD determination, constructed on a commercial SPCE. The sensor was obtained by surface imprinting through electropolymerization using CV and 4-aminobenzoic acid (4-ABA) as a functional monomer. The electrochemical polymerization was studied and optimized. With this approach, the preparation is easy, fast, and green (it uses a small number of reagents). The detection was performed by measuring the DPV signal of the TZD oxidation. An imprinting factor of 71 was reported, and the sensor showed a good selectivity. A linear range of 5–80 µmolL^−1^ and an LOD of 1.6 µmolL^−1^ were reported. The application of the sensor was demonstrated using tap water and human serum samples [60].

Citalopram (CTL) is largely used around de world. It is one of the most important antidepressants of the SSRIs class. Patricia et al. reported selective MIP based electrochemical sensor for CTL determination. In situ polymerization on the surface of the working electrode of an SPCE was acquired through the electropolymerization of 3-amino-4 hydroxybenzoic acid (AHBA) in the presence of CTL. Computational studies, namely molecular dynamics (MD) simulations, were executed to study the polymerization solution and optimize the polymerization conditions to obtain the most efficient MIP formulation. The obtained sensor was characterized by CV and EIS techniques. The sensor showed excellent selectivity in the presence of analog structures and an imprinting factor of 22. The oxidation of CTL was measured by SWV in the 0.1 to 1.25 µmolL^−1^ range with a LOD of 0.162 µmolL^−1^. The validation of the sensor was tested in spiked river water samples [61]. 

Another sensitive and ultra-selective electrochemical sensor for CTL determination was reported by Aminikhah et al. A GCE was firstly modified with hollow nickel nanospheres (hNiNS)/activated MWCNTs@graphene oxide nanoribbons (AMWCNTs@GONRs) composite. This allowed to enhance the electrocatalytic response of CTL, improving the sensitivity. Then in situ electropolymerization was performed using pyrrole as the functional monomer. Several methods were used to characterize the constructed sensor, namely SEM, transmission electron microscopy (TEM), XRD, CV, and EIS. The DPV response to CTL showed two linear dynamic ranges from 0.5 to 10 µmolL^−1^ and 10 to 190 µmolL^−1^ with a LOD of 0.042 µmolL^−1^ and proved to show excellent selectivity towards several compounds. It was successfully applied to measure CTL in urine, serum, and tablet samples [69]. Table 2 summarizes all of the mentioned CMEs, highlighting different aspects of the developed sensors.

## 3. Enzyme-Based Electrochemical Biosensors for the Detection of Antidepressants

An enzyme-modified electrochemical biosensor is an analytic device that incorporates an enzyme as a major part of the processes of recognition and quantification of a compound. This type of enzyme-based reaction involves the ability of the enzyme to catalyze the formation of an electroactive product or quantifiable electric changes on the electrode surface and the analyte when a suitable substrate is present. Enzyme electrodes can be divided into three generations, based on the method of attachment of the enzyme to the base of the transducer: The first generation consists of oxygen-based sensors, the second generation is based on mediator built reactions, and the third generation consists of direct electrochemistry-based sensors [70,71,72]. This third method combines the high specificity advantage of enzymes with the unique advantages of electrochemical detection, such as low cost and high sensitivity, enhancing the ability of the sensor to detect the analyte at lower concentration limits [73].

The selection of the enzymes is the most critical step in the enzymatic biosensors since it provides the selectivity for the biosensor and catalyzes the formation of an electroactive product for detection. Beyond that, the selection of the enzyme immobilization strategy also plays a crucial role, as it determines the characteristics and overall success of the enzyme immobilization process. Enzyme activity, efficient substrate consumption, product formation rate, mass transfer rate, and the cost of the immobilization process influence the selection of immobilization techniques. 

**Figure 7:**
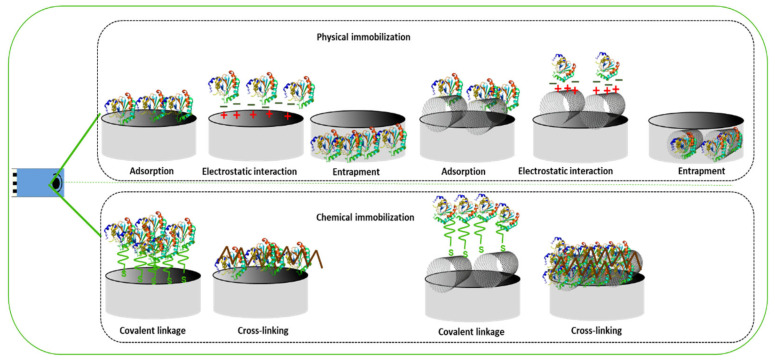
Generic scheme of enzyme immobilization methodologies.

Immobilization methods may be divided into two major categories: Physical and chemical methods. The physical techniques include interactions between the enzyme molecules and the support surfaces via weak bonds, e.g., van der Waals forces, hydrogen bonds, hydrophobic, ionic, and affinity connections. The chemical methods include covalent linkage (either of amide, ether, thioether, or carbamate bonds) or cross-linking between various polymers. Chemical processes are irreversible because the created bonds need a lot of energy to break (Figure 7).

Recently, enzyme-based electrochemical sensors have been developed to detect antidepressant medications [71,72,73,74,75,76,77,78,79,80]. In this case, a drug’s ability to induce the inhibition of certain enzymes is explored, as well as the enzyme’s metabolic capabilities, which permits the development of sensitive sensors capable of monitoring these reactions. The two main enzymes whose properties were explored for the construction of the mentioned electrochemical devices in this review are cytochrome P450 (CYP) enzymes and mono-amine oxidase (MAO), as seen in Table 3.

CYPs constitute a super-family of enzymes with a heme in their active site, being the major agent capable of metabolizing/bioactivating most molecules/drugs, therefore having a huge relevance for clinical pharmacology [74]. Additionally, it is important to note that CYPs are a major source of variability in drug pharmacokinetics and response, increasing the interest in the development of more biosensing-based approaches that can predict individual patients’ responses to certain medications [75]. A major advantage of electrochemical methods in CYPs assays is that they allow direct reduction without the need for electron donors [76]. To improve the efficiency of these CYP-based biosensors, the use of different electrode types and modifications can be employed. 

Iwuoha et al. reported the development of an enzymatic-nanobiosensor based on a reaction mediated by poly (8-anilino-1-napthalene sulphonic acid) (PANSA) nanotubes, with CYP2D6 enzymes, as they constitute SRT binding sites. PANSANTs were prepared through the oxidative polymerization method on a AuE, CYP2D6 enzymes were added to the modified AuE/PANSANTs. SEM methods revealed uniform morphology on the NTs (90 nm in diameter and 600–800 nm in length) that constitute the Au/PANSA/CYP2D6 biosensor. The biosensor’s response to SRT was measured by CV, which revealed a peak potential of the Au/PANSA/CYP2D6 at −315 mV. DPV responses were evaluated in the presence of other CYP2D6 interacting compounds (such as PRX and debrisoquine sulphate) and successfully confirmed that the biosensor contains the CYP2D6 enzymes. The novel sensor achieved a linear response at concentration range of 0.2–1.4 µmolL^−1^, a LOD of 0.13 µmolL^−1^, and was used to detect SRT in pharmaceutical products [77].

Similarly, Ajayi et al. investigated the inhibition of CYP2D6, one of the most relevant isoforms of cytochrome P450, by the antidepressant PRX. PRX was encapsulated in nanostructured poly PANSA and deposited on a gold electrode (Au/PANSA/CYP2D6 biosensor). SEM microscopy studies were performed and revealed a large effective surface for loading large amounts of enzymes in the electrode. Inhibition studies were performed using FLV as a substrate for PRX inhibition, resulting in a decrease in the FLV signal. The electrochemical behavior of the sensor was analyzed by CV and DPV, achieving a linear range in the $5.0–50.0{\;\mathrm{nmolL}}^{-1}$ interval and a LOD of 2.0 nmolL^−1^, as indicated in Table 3 [78].

Monoamine oxidases (MAO) are mitochondrial outer membrane proteins that catalyze the production of hydrogen peroxide through deamination/degradation of various monoamines, such as dopamine, sertraline, and norepinephrine. MAO enzymes have two main isoforms: monoamine oxidase A (MAO-A) and monoamine oxidase B (MAO-B) [79]. One of the main hypotheses that explains depression postulates that MDD is the result of monoamine deficiency. Monoamine oxidase inhibitors (IMAO) have been used for decades and partially support this theory, as they increase monoamine availability [80,81]. The first IMAOs were used as antidepressants, and since then, a variety of molecules capable of MAO inhibition have been discovered and developed into effective medications. In recent years, the growing interest in electrochemical biosensing characteristics, such as low cost and sensitivity, has resulted in the development of MAO sensors capable of monitoring and detecting antidepressants.

Selegiline (SEL) is an irreversible inhibitor of MAO-type B, widely prescribed for Parkinson’s disease and more uncommonly used, at higher doses, for major and atypical depression [82]. Aigner et al. developed a human MAO-type B (hMAO B) biosensor, which could characterize the type of enzyme inhibition and determine inhibitors (like SEL), through the formation of hydrogen peroxide and use of hMAO B substrate phenylethylamine (PEA). For this, SPEs were modified and mixed with manganese dioxide (MnO_2_), a reaction mediator, and hMAO B enzyme. The MnO_2_ ideal concentration was assessed through incubation with two different concentrations of PEA. The researchers found 20% as the ideal concentration. Furthermore, inhibition studies were performed and evaluated the electrochemical effect of different concentrations of PEA: Higher concentrations resulted in lower signals, which proves an irreversible inhibition reaction by SEL. This enzymatic biosensor showed a linear correlation in the 0.51–3.25 µgmL^−1^ SEL hydrochloride interval. Application of the novel sensor to SEL tablets resulted in a mean recovery rate of 105.2%, cementing it as a rapid and useful tool for hMAO B inhibitors detection [83].

Brusnitsyn et al. reported an MAO-based biosensor capable of determining moclobemide (MOC) and AMT in pharmaceutical tablets. The researchers used SPCEs, modified with graphene oxide and MWCNTs functionalized by carboxylic acid. The maximum catalytic activity of the immobilized MAO was observed at pH 7.0, reaffirming that the tested antidepressants have an inhibitory effect on the catalytic activity of the immobilized MAO. A linear concentration range of 10 nmolL^−1^−0.1 mmolL^−1^ was obtained, and the sensor was successfully applied to tablets, as indicated in Table 3 [84]. 

Vela et al. reported an enzymatic biosensor by immobilization, through electro polymerization, of MAO in a polypyrrole polymer, deposited at the surface of a platinum electrode. An interesting characteristic of this study is that the developed biosensor was adapted to a continuous flow injection analysis, with the detection of hydrogen peroxide serving as the analytical signal. The sensor’s LOD was 0.10 mmolL^−1^. The developed methodology was applied in the quantitative analysis of pharmaceutical products containing FLU [85].

Medyantseva et al. have developed several MAO biosensors capable of antidepressant detection. One of the first proposed biosensors by Medyantseva et al. was an amperometric sensor centered on a platinum SPE immobilized with MAO. The proposed methodology could detect three antidepressant medications: Pyrazidol (PYR), petylil (PTL), and FLU. Different monoamines can act as a substrate for MAO. In this study, dopamine was the selected amine, with the electrochemical current of hydrogen peroxide oxidation serving as the analytical signal, as well as the deamination of dopamine. LODs of 0.8 nmolL^−1^, 8.0 nmolL^−1^, and 0.80 µmolL^−1^ were obtained for FLU, PTL, and PYR, respectively. FLU was successfully determined in a pharmaceutical tablet [86].

In another work, Medyantseva et al. explored the capabilities of an amperometric MAO biosensor based on SPCEs, modified with MWCNTs. A LOD of 0.8 nmolL^−1^ was obtained. Additionally, the sensor was tested with moderate success in urine samples [87].

Medyantseva and collaborators expanded on the described biosensor, evaluating the effect of MWCNTs along with silver NPs as surface modifiers of the graphite electrode, with immobilized MAO, for the determination of IMP and AMT in urine samples. It was found that these modifiers improved the sensitivity of the biosensor, with LODs of 7.0 and 8.0 nmolL^−1^ for AMT and IMP, respectively. Finally, the developed biosensor was applied to urine samples containing AMT, with a linear relationship between the concentration of AMT and the analytical signal in the same concentration range as the one verified for the buffer solution [88].

On the other hand, adding to the strategy of analytical signal improvement and nano-surface modification of graphite SPEs, Medyantseva et al. also developed a biosensor capable of detecting AMT, moclobemide, and tianeptine, modified with three types of nanostructured materials: MWCNTs, gold and silver NPs. After the modification, the electrode was coated with MAO. The proposed methodology allowed for the detection of antidepressants in urine and pharmaceutical tablet samples [89].

More recently, Medyantseva’s group reported on an amperometric MAO biosensor, based on the modification of a graphite SPE with nanostructured rGO composites and cobalt NPs by dropwise evaporation. The developed biosensor could detect three different antidepressant drugs: FLU, thioridazine, and tianeptine. Most notably, the addition of the described modifiers significantly improved the analytical characteristics of the sensor, expanding the concentration range from 0.1 mmolL^−1^ to 5 nmolL^−1^. Using tyramine as the substrate of the reaction, FLU, thioridazine, and tianeptine were detected in pharmaceutical tablets, and FLU also being detected in urine [90]. 

**Table 3:** Overview of enzyme-based modified electrode sensors for the detection of antidepressants.

Platform	Enzyme	Inhibitor (Molecule)	Sample	Detection Method	Linear Range	LOD	References
Au/PANSA/CYPD6	CYP2D6	Paroxetine	Pharmaceutical	CV, DPV, SWV	$0.005–0.05{\;µ\mathrm{mol}\;L}^{-1}$	0.002 µmolL^−1^	[78]
Au/PANSA/CYPD6	CYP2D6	Sertraline	Pharmaceutical	CV, DPV, SWV	0.2–1.4 µmolL^−1^	0.13 µmolL^−1^	[77]
SPE-CNTs-GO/MAO	MAO	Moclobemide	Pharmaceutical	CV, AMP	10 nmolL^−1^–0.1 mmolL^−1^	5.0 nmolL^−1^	[84]
SPE-CNTs-GO/MAO	MAO	Amitriptyline	Pharmaceutical	CV, AMP	10 nmolL^−1^–0.1 mmolL^−1^	8.0 nmolL^−1^	[69]
MWCNTs-AgNP-MAO	MAO	Amitriptyline	Pharmaceutical, urine	CV, AMP	10 nmolL^−1^–0.1 mmolL^−1^	8.0 nmolL^−1^	[88]
MWCNTs-AgNP-MAO	MAO	Imipramine	Pharmaceutical, urine	CV, AMP	10 nmolL^−1^–0.1 mmolL^−1^	7.0 nmolL^−1^	[88]
Pt-SPE/MAO r	MAO	Fluoxetine	Pharmaceutical	CV, AMP	1.0 nmolL^−1^–0.1 mmolL^−1^	0.8 nmolL^−1^	[86]
Pt-SPE/MAO r	MAO	Petylyl	Pharmaceutical	CV, AMP	10 nmolL^−1^–0.1 mmolL^−1^	8 nmolL^−1^	[86]
Pt-SPE/MAO r	MAO	Pyrazidol	Pharmaceutical	CV, AMP	0.1 µmolL^−1^–0.1 mmol L^−1^	0.8 µmolL^−1^	[86]
PT-SPE/PPy/MAO	MAO	Fluoxetine	Pharmaceutical	FIA	0.67– 4.33 mmolL^−1^	0.1 mmolL^−1^	[85]
SPE/MWCNT/MAO	MAO	Imipramine	Pharmaceutical, urine	CV	1 × 10^−9^–1 × 10^−4^ molL^−1^	0.8 nmolL^−1^	[87]
MnO_2_/SPE	MAO	Selegeline	Pharmaceuticals	FIA	0.51–3.25 µgmL^−1^	0.15 µgmL^−1^	[83]
SPGE/RGO/CoNPs/MAO	MAO	Fluoxetine	Pharmaceutical, urine	CV	5.0 nmolL^−1^–0.1 mmolL^−1^	0.8 nmolL^−1^	[90]
SPGE/RGO/CoNPs/MAO	MAO	Thioridazine	Pharmaceutical, urine	CV	5.0 nmolL^−1^–0.1 m mmolL^−1^	8.0 nmolL^−1^	[90]
SPGE/RGO/CoNPs/MAO	MAO	Tianeptine	Pharmaceutical, urine	CV	5.0 nmolL^−1^–0.1 m mmolL^−1^	7.0 nmolL^−1^	[90]
SPE/MWCNTs/AuNPAgNP/MAO	MAO	Amitryptiline	Pharmaceutical, urine	CV	--	--	[89]
SPE/MWCNTs/AuNPAgNP/MAO	MAO	Moclobemide	Pharmaceutical, urine	CV	5.0 nmolL^−1^–0.1 mmolL^−1^	0.8 nmolL^−1^	[89]
SPE/MWCNTs/AuNPAgNP/MAO	MAO	Tianeptine	Pharmaceutical, urine	CV	10 nmolL^−1^–0.1 mmolL^−1^	7.0 nmolL^−1^	[89]

Abbreviations: AMP—amperometry; AgNP- silver nanoparticles; Au—gold; AuNP—gold nanoparticles; CNTs—carbon nanotubes; CPY2D6—cytochrome P450 2D6 enzyme; CV—cyclic voltammetry; CoNPs—cobalt nanoparticles; DPV—differential pulse voltammetry; FIA—flow injection analysis; GO—graphene oxide; MAO—monoamine oxidases; MWCNTs—multi-walled carbon nanotubes; MnO_2_—manganese dioxide; PANSA—poly (8-anilino-1-napthalene sulphonic acid); PT—platinum; SPE—screen printed electrode; SWV—square wave voltammetry.

## 4. Conclusions

MDD constitutes one of the main public health challenges of the developed world, with increasing rates of prevalence that impact patients worldwide. Treatment with antidepressants normally diminishes depressive symptoms and establishes good remission rates, but adverse drug reactions, atypical and treatment-resistant depression presentations remain a cause of concern for clinicians. Chromatographic and conventional analytical methods, such as HPLC and GC-MS, are commonly used for the analysis and study of pharmaceutical products, including antidepressant medications. Specialized equipment, highly trained staff, lack of portability, and time-consuming experiments are some of the main drawbacks to these methodologies. Consequently, one of the emerging technologies for the detection of pharmaceutical compounds in the biomedical area that nullifies these characteristics is electrochemical (bio)sensing. Electrochemical (bio)sensors are sensitive, precise, and easy to use and aim to be affordable platforms that allow for real-time monitoring. These devices can be used as pharmaceutical compound detection tools. 

This review paper aimed to assess the use of electrochemical (bio)sensors for the detection of antidepressant drugs, with an emphasis on chemically modified and enzymatic-based biosensors. The majority of the described electrochemical biosensors in the literature for this purpose involve the chemical modification of experimental electrodes. Carbon-based nanomaterials, metal-based nanoparticles, and molecularly imprinted polymers were used for surface modification. Carbon-based nanomaterials, such as CNTs, SWCNTs, and MWCNTs, possess unique characteristics that enhance the electrochemical reaction, whereas metal-based NPs, such as titanium dioxide, improve the sensor’s capabilities and enhance detection. On the other hand, enzymatic-based electrochemical biosensors are incorporated with an enzyme as the biological compound of the electrochemical reaction, CYP-based and MAO-based sensors were the primary types of sensors described in this review.

As a final remark, multidisciplinary efforts from different areas of the research field are essential for the optimization, proper implementation, and improvement of electrochemical (bio)sensors for the detection of pharmaceutical compounds, more specifically antidepressant drugs. In addition, a close relationship between the scientific, research, and industrial areas is vital for the delivery and implementation of these solutions worldwide. Furthermore, with the development of nanotechnology and biotechnology, new biocompatible bionanomaterials have been designed and tested to create an implementable sensor for in vivo monitor analytes and biochemical reactions. 

## Figures and Tables

**Table 1:** Class and chemical structure of the most prescribed antidepressants.

Tricyclic Antidepressant		Selective Serotonin Reuptake Inhibitors	
Antidepressant	Chemical Structure	Antidepressant	Chemical Structure
Amitriptyline	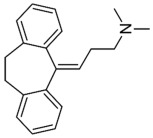	Escitalopram	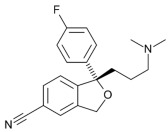
Clomipramine	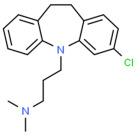	Sertraline	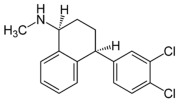
Imipramine	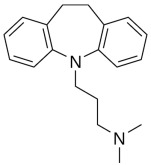	Paroxetine	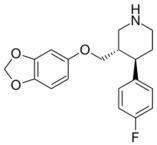
Trimipramine	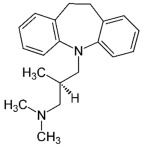	Fluvoxamine	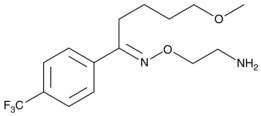
Desipramine	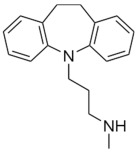	Fluoxetine	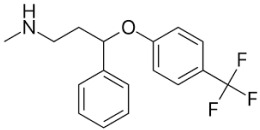
**Monoamine oxidase inhibitor**		**Serotonin antagonist and reuptake inhibitor**	
Selegiline	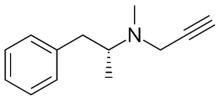	Tradozone	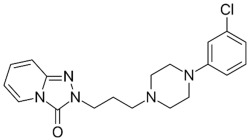
Moclobemide	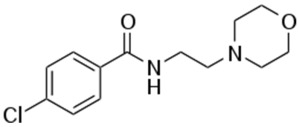	**Serotonin and norepinephrine reuptake inhibitor**	
Pirlindole	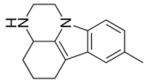	Venlafaxine	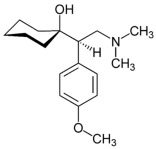

**Table 2:** Overview of chemically modified electrode sensors for the detection of antidepressants.

Antidepressant	Sample	Platform	Technique	Linear Range	LOD	References
Amitriptyline	Pharmaceutical	CNT/PE	CV, DPV, SWV	$0.0–30.0{\;µ\mathrm{molL}}^{-1}$	1.61 µmolL^−1^	[29]
Buzepide Methiodide	Urine, human blood serum	TiO_2_/CPE	CV, DPV	$50{\;\mathrm{nmolL}}^{-1}–50$ µmolL^−1^	8.2 nmolL^−1^	[21]
Clomipramine	Spiked human serum, urine	MWCNTs/GCE	DPV-AAdSV	$14{\;µ\mathrm{molL}}^{-1}–4.5\;$mmol L^−1^	1.315 × 10^−8^ gmL^−1^	[30]
Escitalopram	Urine, cerebrospinal fluid	EGPU-GR	DPV, SWV	1.5–12 µmolL^−1^	0.25 µmolL^−1^ (SWV) 0.32 µmolL^−1^ (DPV)	[33]
Fluvoxamine	Urine, pharmaceuticals	HgNPs/MWCNT/GCE	CV, DPV	0.020–1.750 µmolL^−1^	0.01 µmolL^−1^	[57]
Paroxetine	Urine, pharmaceuticals, blood serum	FCA-CD/CNT/GCE	CV, DPV	$8.0{\;\mathrm{nmolL}}^{-1}–1.0$ µmolL^−1^	0.03 µmolL^−1^	[29]
Imipramine	Urine, pharmaceuticals	FCA-CD/CNT/GCE	CV, DPV	10–350 µmolL^−1^ (CV) 0.1–10 µmolL^−1^ (DPV)	0.03 µmolL^−1^	[29]
Imipramine	Pharmaceuticals	EGRU	CV, SWV	---	4.60 nmolL^−1^	[44]
Sertraline	Human serum	NiLD/AuNPs/MWCNTs/GCE	CV, DPV	0.05–5.50 mmolL^−1^	---	[58]
Sertraline	Spiked plasma	CNT/CsMCPE/SDS	CV, SWV	60.0 nM–15.0 µmolL^−1^	9.2 nmolL^−1^	[37]
Sertraline	Pharmaceuticals, synthetic urine	ZnFe_2_O_4_NP/SPE	CV, DPV	0.07–300 µmolL^−1^	0.02 µmolL^−1^	[55]
Sertraline	Pharmaceuticals, human serum	MIP/Gr/SPE	CV, DPV	5.0 nmolL^−1^–0.75 µmol L^−1^	1.99 nmolL^−1^	[67]
Fluoxetine	Pharmaceuticals, plasms	Graphene/MIP/CPE	DPV	6 nmolL^−1^–0.1 µmolL^−1^	2.8 nmol L^−1^	[59]
Trimipramine	Human blood and serum	MWCNT/MIP/CPE	SWV	0.10–25 nmolL^−1^	0.045 nmolL^−1^	[61]
Trazodone	Tap water, human serum	MIP/SPCE	CV, DPV	5–80 µmolL^−1^	1.6 µmolL^−1^	[60]
Citalopram	Spiked river water	MIP/SPCE	CV, EIS	0.10–1.25 µmolL^−1^	0.162 µmolL^−1^	[61]
Citalopram	Pharmaceuticals, urine, serum	AMWCNTs@GONRs	CV, EIS, DPV	0.5–10 µmolL^−1^	0.042 µmolL^−1^	[69]
Trimipramine	Plasma, urine	MWCNT/CPE	DPV	----	0.002 µmolL^−1^	[31]
Venlafaxine	Pharmaceuticals	MWCNT/IL/GCE	CV, SWV	2.0 µmolL^−1^–2.0 mmol L^−1^	1.69 µmolL^−1^	[32]
Venlafaxine	Serum	CoPd@Al_2_O_3_/GCE	CV, SWASV	1.95 mmolL^−1^–0.5 µmolL^−1^	1.96 pmolL^−1^	[55]
Venlafaxine	Serum, pharmaceuticals	NiC_O2_O_4_/rGO/GCE	CV, SWV	5.0–500 nmolL^−1^	3.4 nmolL^−1^	[46]

Abbreviations: AAdSV—anodic adsorptive stripping voltammetry; AMWCNTs—activated multi-walled carbon nanotubes; AuNPS—gold nanoparticles; CNTs- carbon nanotubes; CPE—carbon paste electrode; CV—cyclic voltammetry; CoPd@Al_2_O_3_—palladium cobalt/aluminum dioxide; Cs—cesium; DPV—differential pulse voltammetry; EGPU—graphite-polyurethane electrode; EIS—electrochemical impedance spectroscopy; FCA-CD—ferrocene carboxylic acid-β-cyclodextrin; GCE—glassy carbon electrode; GONRs—graphene oxide nanoribbons; GR—graphene; HgNPs—mercury nanoparticles; MIP—molecularly imprinted polymer; MWCNTs—multi-walled carbon nanotubes; NiCO_2_O_4_—nickel cobalt oxide; NiLD—electropolymerized nanostructured levodopa film; SDS—dodecyl sulphate; SWASV—square-wave anodic stripping voltammetry; SWV—square wave voltammetry; TiO_2_—titanium dioxide; ZnFe_2_O_4_NPs—zinc ferrite nanoparticles; rGO—reduced graphene oxide.
